# Supplementary material for: Effect of Fractionation and Processing Conditions on the Digestibility of Plant Proteins as Food Ingredients
Source: Foods. 2022 Mar 18;11(6):870. doi: 10.3390/foods11060870 (PMC8955167; doi:10.3390/foods11060870)
Supplement: Supplementary file 1 [file foods-11-00870-s001.zip › foods-1628852-supplementary.pdf]

Supplementary material

Effect of fractionation and processing conditions on the digestibility of plant proteins as food ingredients

Andrea Rivera del Rio, Remko M. Boom and Anja E.M. Janssen

Table S1. Summary of digestion assays, sample treatment and measurement from studies investigating the effect of processing on protein digestion.

Crop	Digestion assay gastric	Digestion assay small intestinal	Digestion sample treatment/measurement	Effect	Reference
Pre-fractionation treatments					
High temperature soaking					
soybean	3 h	N/A	TCA-soluble nitrogen	**h**	Wally-Vallim, *et al.* [1]
cowpea	2 h	2 h pancreatin	supernatant, ultrafiltered protein cutoff 3 kDa<br>TCA-soluble nitrogen defined as DH	**h**	Marques, *et al.* [2]
Germination					
soybean	2 h	24 h trypsin	TCA-soluble nitrogen	**h**	Dikshit and Ghadle [3]
black bean	1 h	2 h pancreatin	supernatant<br>DH by ninhydrin reaction<br>Samples digested for 24 h considered as 100% DH	**h**	López-Barrios, *et al.* [4]
soybean	N/A	trypsin (time not specified)	DH by OPA reaction	**hi**	Aijie, *et al.* [5]
black soybean	30 min	30 min pancreatin	supernatant: molecular weight distribution	lower proportion of <0.15 kDa peptides in germinated samples	Sefatie, *et al.* [6]
Fermentation					
lupin	N/A	10 min trypsin, α-	pH drop	**h**	Bartkiene, *et al.* [7]

		chymotrypsin and serine-type protease			
Conventional protein fractionation					
soybean		*in vivo* rat assay	PDCAAS	=	Hughes, *et al.* [8]
soybean		*in vivo* pig assay	Standard ileal digestibility	=	Pedersen, *et al.* [9]
soybean	3 h	N/A	TCA-soluble nitrogen	=	Da Silva Pinto, *et al.* [10]
Alkaline treatment					
rice	2 h	2 h trypsin and *in vivo* rat assay	TCA-soluble nitrogen and absorption	i	Zhang, *et al.* [11]
lupin	N/A	up to 28 h trypsin and chymotrypsin	TCA-soluble nitrogen	i	Yu, *et al.* [12]
quinoa	6 h	N/A	DH by OPA reaction<br>Size-exclusion chromatography	i	Ruiz, *et al.* [13]
Alternative protein fractionation					
'alternative' chemicals					
soybean	2 h	24 h pancreatin	TCA-soluble nitrogen	=	Chamba, *et al.* [14]
lupin	N/A	10 min trypsin, chymotrypsin and peptidase	pH drop	**h**	Lqari, *et al.* [15]
chickpea	N/A	10 min trypsin, α-chymotrypsin and peptidase	pH drop	=	Sánchez-Vioque, *et al.* [16]
brown lentil	1 h	24 h pancreatin	Free amino groups by TNBS reaction	=	Joehnke, *et al.* [17]
alternative drying methods					
hempseed	N/A	10 min trypsin, α-chymotrypsin and protease	pH drop	freeze>oven> vacuum oven drying	Lin, *et al.* [18]
buckwheat	2 h	2 h trypsin	TCA-soluble nitrogen release and SDS-PAGE	freeze>spray drying	Tang [19]
enzyme assisted fractionation					
soybean	(not specified)	N/A	nitrogen solubility	=	De Almeida, *et al.* [20]
white sorghum	2 h	N/A	nitrogen solubility	=	De Mesa-Stonestreet, *et al.* [21]
air classification					

pea, fava bean and lentil	*in vivo* mouse assay		protein digestibility	i	Bhatty and Christison [22]
fava bean	1 h	24 h pancreatin	Free amino groups by TNBS reaction	i	Vogelsang-O'Dwyer, *et al.* [23]
quinoa	6 h	N/A	DH by OPA reaction<br>Size-exclusion chromatography	**h**	Opazo-Navarrete, *et al.* [24]
Post-fractionation processing					
Fermentation					
yellow pea	N/A	10 min trypsin, α-chymotrypsin and protease	pH drop<br>in vitro PDCAAS	**h**<br>(lower IVPDCAAS)	Çabuk, *et al.* [25]
soybean	soft rat gastrointestinal model with dynamic gastric and duodenal (pancreatin) parts		TCA-soluble free-amino acids	**h**	Huang, *et al.* [26]
Ultrasound					
fava bean	N/A	10 min trypsin, α-chymotrypsin and peptidase	pH drop	i	Martínez-Velasco, *et al.* [27]
Proteolysis					
soybean	30 min	30 min chymotrypsin (non-sequential)	TCA-soluble nitrogen	**h**	Ge and Zhang [28]
soybean	N/A	2 h trypsin and α-chymotrypsin	pH drop and free amino groups by ninhydrin reaction	**=**	Nguyen, *et al.* [29]
soybean and zein	30 min	2 h pancreatin	Tricine-SDS PAGE	**=** soy, **h** zein	Kaur, *et al.* [30]
soybean, wheat gluten and zein	*in vivo* rat assay		true ileal digestibility	**=** soy, wheat gluten, **h** zein	Rutherfurd, *et al.* [31]
soybean	*in vivo* rat assay		true gastric total protein digestion	**h**	Montoya, *et al.* [32]
green lentil	N/A	10 min trypsin, α-chymotrypsin and peptidase	pH drop and size-exclusion chromatography	i	Aryee and Boye [33]
rapeseed	*in vivo* rat assay<br>*in vivo* human assay		true digestibility<br>PDCAAS	**h**	Fleddermann, *et al.* [34]
rice bran	2 h	2 h pancreatin	TCA-soluble nitrogen	**h**	Singh, *et al.* [35]
chickpea	N/A	10 min trypsin, α-chymotrypsin and peptidase	pH drop	**=**	Clemente, *et al.* [36]

chickpea	N/A	11 min trypsin, α-chymotrypsin and protease	pH drop	**=**	Goertzen, *et al.* [37]
Heat treatment					
soybean	1 h	N/A	DH by OPA reaction	**hi**	Tian, *et al.* [38]
soybean	1 h	3 h pancreatin	TCA-soluble nitrogen and SDS PAGE	**h**	Chen, *et al.* [39]
soybean	1 h	2 h trypsin	DH by OPA reaction	**h**	Ren, *et al.* [40]
soybean	Infogest 1.0		TCA-soluble nitrogen	**h**	Zhang, *et al.* [41]
soybean	*in vivo* rat assay		Apparent digestibility from faeces	**i**	Kim and Barbeau [42]
lupin	N/A	10 min trypsin, α-chymotrypsin and peptidase	pH drop	**h**	Sathe, *et al.* [43]
winged bean	N/A	10 min trypsin, α-chymotrypsin and peptidase	pH drop	**h**	Sathe, *et al.* [44]
red bean, red kidney bean and mung bean	2 h	2 h trypsin	TCA-soluble nitrogen	i red bean,<br>= red kidney bean<br>**h**i mung bean	Tang, *et al.* [45]
soybean and pea	2 h	N/A	DH by OPA reaction and size-exclusion chromatography	**=**	Rivera del Rio, *et al.* [46]
quinoa	6 h	N/A	DH by OPA reaction and size-exclusion chromatography	**i**	Opazo-Navarrete, *et al.* [47]
lupin	30 min	N/A	<3 kDa peptides from size-exclusion chromatography	**= i**	Pelgrom, *et al.* [48]
navy bean	N/A	24 h trypsin, α-chymotrypsin and peptidase	DH by TNBS reaction	**h**	Sathe, *et al.* [49]
sweet potato	*in vivo* rat assay		PDCAAS	**h**<br>autoclaved> microwaved> dry heated>native	Sun, *et al.* [50]
lentil	N/A	2 h trypsin and chymotrypsin	DH by TNBS reaction	**h**	Neves and Lourenço [51]
chickpea	2 h	2 h trypsin and chymotrypsin or 10 min trypsin, chymotrypsin and peptidase	DH by TNBS reaction and pH drop	**h**	Tavano and Neves [52]
white common bean	*in vivo* rat assay		absorption or protein intestinal digestibility	i	Carbonaro, *et al.* [53]

and fava bean					
navy bean	not specified		*"in vitro protein digestibility"*	**hi**	Chang and Satterlee [54]
soybean	N/A	10 min trypsin, α-chymotrypsin and peptidase	pH drop	i	Wu, *et al.* [55]
soybean and rapeseed	30 min	pancreatin and diffusion through a 1000 MWCO membrane	nitrogen and amino acid release in dialysates	i	Savoie, *et al.* [56]
soybean	*in vivo* rat assay		true protein digestibility	i	Sarwar, *et al.* [57]
High pressure processing					
yellow pea	Infogest 1.0		SDS PAGE and pH stat	**h**	Laguna, *et al.* [58]
Modifications					
Transglutaminase-catalysed polymerization					
red kidney bean	N/A	2 h trypsin	TCA-soluble nitrogen	i	Yin, *et al.* [59]
Phaseolus vulgaris L. bean	1 h	1 h trypsin (non-sequential)	SDS PAGE	i	Mariniello, *et al.* [60]
soybean	2 h	2 h trypsin	TCA-soluble nitrogen	i	Tang, *et al.* [61]
red kidney bean	N/A	2 h trypsin	TCA-soluble nitrogen	**h**	Tang, *et al.* [62]
soybean	1 h	3 h pancreatin	SDS PAGE	i	Li and Damodaran [63]
soybean	2 h or 1 h for sequential digestion	1 h trypsin	TCA-soluble nitrogen	i	Sheng and Zhao [64]
soybean	time not specified	trypsin (time not specified)	TCA-soluble nitrogen	**h**	Fu and Zhao [65]
soybean	2 h or 1 h for sequential digestion	1 h trypsin	TCA-soluble nitrogen	**h**	Zhu, *et al.* [66]
soybean	N/A	10 min trypsin, α-chymotrypsin and peptidase	pH drop	i	Gan, *et al.* [67]
Acylation					
red kidney bean	N/A	2 h trypsin	TCA-soluble nitrogen	**h**	Yin, *et al.* [68]
mung bean	N/A	trypsin and pancreatin	pH stat	**h**	El-Adawy [69]

		(time not specified)			
soybean	N/A	10 min trypsin, α-chymotrypsin and peptidase	pH drop	**h**	Achouri and Zhang [70]
soybean	*in vivo* rat assay		protein efficiency ratio	**h** =	de Regil and Calderón de la Barca [71]
Complexation with phenolic compounds					
soybean	2 h	24 h trypsin and chymotrypsin	size exclusion chromatography	=	Budryn, *et al.* [72]
soybean	time not specified	trypsin and chymotrypsin (time not specified)	TCA-soluble nitrogen	i	Rohn, *et al.* [73]
	in vivo rat assay		nitrogen efficiency<br>true nitrogen digestibility<br>PDCAAS		
yellow pea	3 h	4 h pancreatin	Free amino groups by TNBS reaction	i	Strauch and Lila [74]
soybean	1 h	2 h trypsin	SDS PAGE	i	Yang, *et al.* [75]
Other modifications					
soybean	1 h	2 h pancreatin	SDS PAGE and free amino acids	i	Chen, *et al.* [76]
soybean, cottonseed and peanut	N/A	10 min trypsin, α-chymotrypsin and peptidase	pH drop	i	Rhee and Rhee [77]
soybean	2 h	2 h pancreatin	TCA-soluble nitrogen	**h**	Wang, *et al.* [78]
Protein oxidation					
soybean	Infogest 1.0	N/A	DH by OPA reaction	**hi**	Zhao, *et al.* [79]
soybean	1 h	2 h pancreatin	SDS PAGE	i	Chen, *et al.* [80]
soybean	3 h	N/A	DH by OPA reaction	=	Duque-Estrada, *et al.* [81]
Structure formation					
Gelling					
soybean and yellow pea	3 h	N/A	DH by OPA reaction and size-exclusion chromatography	i soybean<br>= yellow pea	Opazo-Navarrete, *et al.* [82]
lentil and fava bean	Infogest 1.0		ultrafiltration through 50 kDa, 30 kDa, and 10 kDa Mw cut-offs<br>SDS PAGE	=	Hall and Moraru [83]
soybean	1 h	2 h pancreatin	pH stat	**h**	Rui, *et al.* [84]
soybean	2 h	N/A	TCA-soluble nitrogen	i	Ou, *et al.* [85]
Extrusion and texturization					

soybean	N/A	10 min trypsin, α-chymotrypsin and peptidase	pH drop	**h**	Omosebi, *et al.* [86]
yellow pea	N/A	10 min trypsin, α-chymotrypsin and peptidase	pH drop	**h**	Wang, *et al.* [87]
rice	1 h	2 h pancreatin	fluorescence spectral analysis	i	Li, *et al.* [88]
Intragastic gelling					
soybean	2 h	N/A	SDS PAGE of aqueous phase	(	Hu, *et al.* [89]
soybean	Infogest 1.0	N/A	SDS PAGE	(	Huang, *et al.* [90]
Animal-plant hybrid foods					
soybean, rice and yellow pea	1 h	4 h trypsin and chymotrypsin	TCA-soluble nitrogen, amine content by ninhydrin reaction, in vitro PDCAAS	**h**	Khalesi and Fitzgerald [91]
yellow pea, rice and lentil	Infogest 2.0		TCA-soluble free amino acids	i	Baugreet, *et al.* [92]
Nutrient interactions					
yellow pea	2 h	2 h pancreatin	SDS PAGE	i	Oñate Narciso and Brennan [93]
chickpea	N/A	10 min trypsin, α-chymotrypsin and peptidase	pH drop	i	Sánchez-Vioque, *et al.* [94]

DH, degree of hydrolysis; N/A, not applicable; OPA, o-phthalaldehyde; PDCAAS, protein digestibility-corrected amino acid score; SDS PAGE, sodium dodecyl sulphate–polyacrylamide gel electrophoresis; TCA, trichloroacetic acid; TNBS, trinitrobenzenesulfonic acid. The pH drop method normally refers to the method by Hsu, *et al.* [95].

References

1. Wally-Vallim, A.P.; Vanier, N.L.; da Rosa Zavareze, E.; Zambiazi, R.C.; de Castro, L.A.S.; Schirmer, M.A.; Elias, M.C. Isoflavone Aglycone Content and the Thermal, Functional, and Structural Properties of Soy Protein Isolates Prepared from Hydrothermally Treated Soybeans. *Journal of Food Science* **2014**, *79*, E1351-E1358, doi:10.1111/1750-3841.12506.
2. Marques, M.R.; Soares Freitas, R.A.M.; Corrêa Carlos, A.C.; Siguemoto, É.S.; Fontanari, G.G.; Arêas, J.A.G. Peptides from cowpea present antioxidant activity, inhibit cholesterol synthesis and its solubilisation into micelles. *Food Chemistry* **2015**, *168*, 288-293, doi:10.1016/j.foodchem.2014.07.049.
3. Dikshit, M.; Ghadle, M. Effect of sprouting on nutrients, antinutrients and in vitro digestibility of the MACS-13 soybean variety. *Plant Foods for Human Nutrition* **2003**, *58*, 1-11, doi:10.1023/B:QUAL.0000040357.70606.4c.
4. López-Barrios, L.; Antunes-Ricardo, M.; Gutiérrez-Uribe, J.A. Changes in antioxidant and antiinflammatory activity of black bean (Phaseolus vulgaris L.) protein isolates due to germination and enzymatic digestion. *Food Chemistry* **2016**, *203*, 417-424, doi:10.1016/j.foodchem.2016.02.048.
5. Aijie, L.; Shouwei, Y.; Li, L. Structure, trypsin inhibitor activity and functional properties of germinated soybean protein isolate. *International Journal of Food Science and Technology* **2014**, *49*, 911-919, doi:10.1111/ijfs.12386.
6. Sefatie, R.S.; Fatoumata, T.; Eric, K.; Shi, Y.H.; Guo-Wei, L. In vitro antioxidant activities of protein hydrolysate from germinated black soybean (Glycine max L.). *Advance Journal of Food Science and Technology* **2013**, *5*, 453-459, doi:10.19026/ajfst.5.3290.

7. Bartkiene, E.; Sakiene, V.; Bartkevics, V.; Rusko, J.; Lele, V.; Juodeikiene, G.; Wiacek, C.; Braun, P.G. Lupinus angustifolius L. lactofermentation and protein isolation: effects on phenolic compounds and genistein, antioxidant properties, trypsin inhibitor activity, and protein digestibility. *European Food Research and Technology* **2018**, *244*, 1521-1531, doi:10.1007/s00217-018-3066-8.
8. Hughes, G.J.; Ryan, D.J.; Mukherjea, R.; Schasteen, C.S. Protein digestibility-corrected amino acid scores (PDCAAS) for soy protein isolates and concentrate: Criteria for evaluation. *Journal of Agricultural and Food Chemistry* **2011**, *59*, 12707-12712, doi:10.1021/jf203220v.
9. Pedersen, C.; Almeida, J.S.; Stein, H.H. Analysis of published data for standardized ileal digestibility of protein and amino acids in soy proteins fed to pigs. *Journal of Animal Science* **2016**, *94*, 340-343, doi:10.2527/jas2015-9864.
10. Da Silva Pinto, M.; Lajolo, F.M.; Genovese, M.I. Effect of storage temperature and water activity on the content and profile of isoflavones, antioxidant activity, and in vitro protein digestibility of soy protein isolates and defatted soy flours. *Journal of Agricultural and Food Chemistry* **2005**, *53*, 6340-6346, doi:10.1021/jf0502451.
11. Zhang, Z.; Wang, Y.; Li, Y.; Dai, C.; Ding, Q.; Hong, C.; He, Y.; He, R.; Ma, H. Effect of alkali concentration on digestibility and absorption characteristics of rice residue protein isolates and lysinoalanine. *Food Chemistry* **2019**, *289*, 609-615, doi:10.1016/j.foodchem.2019.03.085.
12. Yu, R.S.T.; Kyle, W.S.A.; Hung, T.V.; Zeckler, R. Characterisation of aqueous extracts of seed proteins of Lupinus albus and Lupinus angustifolius. *Journal of the Science of Food and Agriculture* **1987**, *41*, 205-218, doi:10.1002/jsfa.2740410303.
13. Ruiz, G.A.; Opazo-Navarrete, M.; Meurs, M.; Minor, M.; Sala, G.; van Boekel, M.; Stieger, M.; Janssen, A.E.M. Denaturation and in Vitro Gastric Digestion of Heat-Treated Quinoa Protein Isolates Obtained at Various Extraction pH. *Food Biophysics* **2016**, *11*, 184-197, doi:10.1007/s11483-016-9429-4.
14. Chamba, M.V.M.; Hua, Y.; Murekatete, N.; Chen, Y. Effects of synthetic and natural extraction chemicals on yield, composition and protein quality of soy protein isolates extracted from full-fat and defatted flours. *Journal of Food Science and Technology* **2013**, *52*, 1016-1023, doi:10.1007/s13197-013-1084-x.
15. Lqari, H.; Vioque, J.; Pedroche, J.; Millán, F. Lupinus angustifolius protein isolates: chemical composition, functional properties and protein characterization. *Food Chemistry* **2002**, *76*, 349-356, doi:10.1016/S0308-8146(01)00285-0.
16. Sánchez-Vioque, R.; Clemente, A.; Vioque, J.; Bautista, J.; Millán, F. Protein isolates from chickpea (Cicer arietinum L.): Chemical composition, functional properties and protein characterization. *Food Chemistry* **1999**, *64*, 237-243, doi:10.1016/s0308-8146(98)00133-2.
17. Joehnke, M.S.; Jeske, S.; Ispiryan, L.; Zannini, E.; Arendt, E.K.; Bez, J.; Sørensen, J.C.; Petersen, I.L. Nutritional and anti-nutritional properties of lentil (Lens culinaris) protein isolates prepared by pilot-scale processing. *Food Chemistry: X* **2021**, *9*, doi:10.1016/j.fochx.2020.100112.
18. Lin, Y.; Pangloli, P.; Dia, V.P. Physicochemical, functional and bioactive properties of hempseed (Cannabis sativa L.) meal, a co-product of hempseed oil and protein production, as affected by drying process. *Food Chemistry* **2021**, *350*, doi:10.1016/j.foodchem.2021.129188.
19. Tang, C.H. Functional properties and in vitro digestibility of buckwheat protein products: Influence of processing. *Journal of Food Engineering* **2007**, *82*, 568-576, doi:10.1016/j.jfoodeng.2007.01.029.
20. De Almeida, N.M.; De Moura Bell, J.M.L.N.; Johnson, L.A. Properties of soy protein produced by countercurrent, two-stage, enzyme-assisted aqueous extraction. *JAOCS, Journal of the American Oil Chemists' Society* **2014**, *91*, 1077-1085, doi:10.1007/s11746-014-2436-z.
21. De Mesa-Stonestreet, N.J.; Alavi, S.; Gwirtz, J. Extrusion-enzyme liquefaction as a method for producing sorghum protein concentrates. *Journal of Food Engineering* **2012**, *108*, 365-375, doi:10.1016/j.jfoodeng.2011.07.024.
22. Bhatty, R.S.; Christison, G.I. Composition and nutritional quality of pea (Pisum sativum L.), faba bean (Vicia faba L. spp. minor) and lentil (Lens culinaris Medik.) meals, protein concentrates and isolates. *Qualitas Plantarum Plant Foods for Human Nutrition* **1984**, *34*, 41-51, doi:10.1007/bf01095071.
23. Vogelsang-O'Dwyer, M.; Petersen, I.L.; Joehnke, M.S.; Sørensen, J.C.; Bez, J.; Detzel, A.; Busch, M.; Krueger, M.; O'Mahony, J.A.; Arendt, E.K.; et al. Comparison of Faba bean protein ingredients produced using dry fractionation and isoelectric precipitation: Techno-functional, nutritional and environmental performance. *Foods* **2020**, *9*, doi:10.3390/foods9030322.
24. Opazo-Navarrete, M.; Schutyser, M.A.I.; Boom, R.M.; Janssen, A.E.M. Effect of pre-treatment on in vitro gastric digestion of quinoa protein (Chenopodium quinoa Willd.) obtained by wet and dry fractionation. *International Journal of Food Sciences and Nutrition* **2018**, *69*, 1-11, doi:10.1080/09637486.2017.1332171.
25. Çabuk, B.; Nosworthy, M.G.; Stone, A.K.; Korber, D.R.; Tanaka, T.; House, J.D.; Nickerson, M.T. Effect of fermentation on the protein digestibility and levels of non-nutritive compounds of pea protein concentrate. *Food Technology and Biotechnology* **2018**, *56*, 257-264, doi:10.17113/ftb.56.02.18.5450.
26. Huang, J.; Liu, Z.; Rui, X.; L'Hocine, L.; Zhang, Q.; Li, W.; Dong, M. Assessment of the effect of lactic acid fermentation on the gastroduodenal digestibility and immunoglobulin e binding capacity of soy proteins: Via an in vitro dynamic gastrointestinal digestion model. *Food and Function* **2020**, *11*, 10467-10479, doi:10.1039/d0fo02023k.
27. Martínez-Velasco, A.; Lobato-Calleros, C.; Hernández-Rodríguez, B.E.; Román-Guerrero, A.; Alvarez-Ramirez, J.; Vernon-Carter, E.J. High intensity ultrasound treatment of faba bean (Vicia faba L.) protein: Effect on surface properties, foaming ability and structural changes. *Ultrasonics Sonochemistry* **2018**, *44*, 97-105, doi:10.1016/j.ultsonch.2018.02.007.
28. Ge, S.J.; Zhang, L.X. Predigestion of soybean proteins with immobilized trypsin for infant formula. *Applied Biochemistry and Biotechnology* **1993**, *43*, 199-209, doi:10.1007/bf02916453.
29. Nguyen, T.T.P.; Bhandari, B.; Cichero, J.; Prakash, S. In vitro digestion of infant formulations with hydrolysed and non-hydrolysed proteins from dairy and soybean. *Food and Function* **2016**, *7*, 4908-4919, doi:10.1039/c6fo01240j.
30. Kaur, L.; Rutherfurd, S.M.; Moughan, P.J.; Drummond, L.; Boland, M.J. Actinidin enhances protein digestion in the small intestine as assessed using an in vitro digestion model. *Journal of Agricultural and Food Chemistry* **2010**, *58*, 5074-5080, doi:10.1021/jf903835g.
31. Rutherfurd, S.M.; Montoya, C.A.; Zou, M.L.; Moughan, P.J.; Drummond, L.N.; Boland, M.J. Effect of actinidin from kiwifruit (Actinidia deliciosa cv. Hayward) on the digestion of food proteins determined in the growing rat. *Food Chemistry* **2011**, *129*, 1681-1689, doi:10.1016/j.foodchem.2011.06.031.
32. Montoya, C.A.; Hindmarsh, J.P.; Gonzalez, L.; Boland, M.J.; Moughan, P.J.; Rutherfurd, S.M. Dietary actinidin from kiwifruit (Actinidia deliciosa cv. Hayward) increases gastric digestion and the gastric emptying rate of several dietary proteins in growing rats. *Journal of Nutrition* **2014**, *144*, 440-446, doi:10.3945/jn.113.185744.
33. Aryee, A.N.A.; Boye, J.I. Improving the Digestibility of Lentil Flours and Protein Isolate and Characterization of Their Enzymatically Prepared Hydrolysates. *International Journal of Food Properties* **2016**, *19*, 2649-2665, doi:10.1080/10942912.2015.1123269.

34. Fleddermann, M.; Fechner, A.; Rößler, A.; Bähr, M.; Pastor, A.; Liebert, F.; Jahreis, G. Nutritional evaluation of rapeseed protein compared to soy protein for quality, plasma amino acids, and nitrogen balance - A randomized cross-over intervention study in humans. *Clinical Nutrition* **2013**, *32*, 519-526, doi:10.1016/j.clnu.2012.11.005.
35. Singh, T.P.; Siddiqi, R.A.; Sogi, D.S. Enzymatic modification of rice bran protein: Impact on structural, antioxidant and functional properties. *LWT* **2021**, *138*, doi:10.1016/j.lwt.2020.110648.
36. Clemente, A.; Vioque, J.; Sánchez-Vioque, R.; Pedroche, J.; Bautista, J.; Millán, F. Protein quality of chickpea (Cicer arietinum L.) protein hydrolysates. *Food Chemistry* **1999**, *67*, 269-274, doi:10.1016/s0308-8146(99)00130-2.
37. Goertzen, A.D.; House, J.D.; Nickerson, M.T.; Tanaka, T. The impact of enzymatic hydrolysis using three enzymes on the nutritional properties of a chickpea protein isolate. *Cereal Chemistry* **2021**, *98*, 275-284, doi:10.1002/cche.10361.
38. Tian, T.; Teng, F.; Zhang, S.; Qi, B.; Wu, C.; Zhou, Y.; Li, L.; Wang, Z.; Li, Y. A Study of Structural Change During In Vitro Digestion of Heated Soy Protein Isolates. *Foods (Basel, Switzerland)* **2019**, *8*, 594, doi:10.3390/foods8120594.
39. Chen, F.P.; Li, B.S.; Tang, C.H. Nanocomplexation between Curcumin and Soy Protein Isolate: Influence on Curcumin Stability/Bioaccessibility and in Vitro Protein Digestibility. *Journal of Agricultural and Food Chemistry* **2015**, *63*, 3559-3569, doi:10.1021/acs.jafc.5b00448.
40. Ren, C.; Xiong, W.; Peng, D.; He, Y.; Zhou, P.; Li, J.; Li, B. Effects of thermal sterilization on soy protein isolate/polyphenol complexes: Aspects of structure, in vitro digestibility and antioxidant activity. *Food Research International* **2018**, *112*, 284-290, doi:10.1016/j.foodres.2018.06.034.
41. Zhang, Y.; Chen, S.; Qi, B.; Sui, X.; Jiang, L. Complexation of thermally-denatured soybean protein isolate with anthocyanins and its effect on the protein structure and in vitro digestibility. *Food Research International* **2018**, *106*, 619-625, doi:10.1016/j.foodres.2018.01.040.
42. Kim, Y.A.; Barbeau, W.E. Changes in the nutritive value of soy protein concentrate during autoclaving. *Plant Foods for Human Nutrition* **1991**, *41*, 179-192, doi:10.1007/bf02194086.
43. Sathe, S.K.; Deshpande, S.S.; Salunkhe, D.K. Functional Properties of Lupin Seed (Lupinus mutabilis) Proteins and Protein Concentrates. *Journal of Food Science* **1982**, *47*, 491-497, doi:10.1111/j.1365-2621.1982.tb10110.x.
44. Sathe, S.K.; Deshpande, S.S.; Salunkhe, D.K. Functional Properties of Winged Bean [Psophocarpus tetragonolobus (L.) DC] Proteins. *Journal of Food Science* **1982**, *47*, 503-509, doi:10.1111/j.1365-2621.1982.tb10112.x.
45. Tang, C.H.; Chen, L.; Ma, C.Y. Thermal aggregation, amino acid composition and in vitro digestibility of vicilin-rich protein isolates from three Phaseolus legumes: A comparative study. *Food Chemistry* **2009**, *113*, 957-963, doi:10.1016/j.foodchem.2008.08.038.
46. Rivera del Rio, A.; Opazo-Navarrete, M.; Cepero-Betancourt, Y.; Tabilo-Munizaga, G.; Boom, R.M.; Janssen, A.E.M. Heat-induced changes in microstructure of spray-dried plant protein isolates and its implications on in vitro gastric digestion. *LWT* **2020**, *118*, doi:10.1016/j.lwt.2019.108795.
47. Opazo-Navarrete, M.; Tagle Freire, D.; Boom, R.M.; Janssen, A.E.M. The Influence of Starch and Fibre on In Vitro Protein Digestibility of Dry Fractionated Quinoa Seed (Riobamba Variety). *Food Biophysics* **2019**, *14*, 49-59, doi:10.1007/s11483-018-9556-1.
48. Pelgrom, P.J.; Berghout, J.A.; van der Goot, A.J.; Boom, R.M.; Schutyser, M.A. Preparation of functional lupine protein fractions by dry separation. *LWT-Food Science and Technology* **2014**, *59*, 680-688, doi:10.1016/j.lwt.2014.06.007.
49. Sathe, S.K.; Iyer, V.; Salunkhe, D.K. Functional Properties of the Great Northern Bean (Phaseolus Vulgaris L.) Proteins. Amino Acid Composition, In Vitro Digestibility, and Application to Cookies. *Journal of Food Science* **1982**, *47*, 8-11, doi:10.1111/j.1365-2621.1982.tb11014.x.
50. Sun, M.; Mu, T.; Zhang, M.; Arogundade, L.A. Nutritional assessment and effects of heat processing on digestibility of Chinese sweet potato protein. *Journal of Food Composition and Analysis* **2012**, *26*, 104-110, doi:10.1016/j.jfca.2012.03.008.
51. Neves, V.A.; Lourenço, E.J. Isolation and in vitro hydrolysis of globulin G1 from lentils (lens culinaris, medik). *Journal of Food Biochemistry* **1995**, *19*, 109-120, doi:10.1111/j.1745-4514.1995.tb00525.x.
52. Tavano, O.L.; Neves, V.A. Isolation, solubility and in vitro hydrolysis of chickpea vicilin-like protein. *LWT - Food Science and Technology* **2008**, *41*, 1244-1251, doi:10.1016/j.lwt.2007.08.003.
53. Carbonaro, M.; Grant, G.; Cappelloni, M. Heat-induced denaturation impairs digestibility of legume (Phaseolus vulgaris L and Vicia faba L) 7S and 11S globulins in the small intestine of rat. *Journal of the Science of Food and Agriculture* **2005**, *85*, 65-72, doi:10.1002/jsfa.1940.
54. Chang, K.C.; Satterlee, L.D. Isolation and Characterization of the Major Protein from Great Northern Beans (Phaseolus vulgaris). *Journal of Food Science* **1981**, *46*, 1368-1373, doi:10.1111/j.1365-2621.1981.tb04177.x.
55. Wu, W.; Hettiarachchy, N.S.; Kalapathy, U.; Williams, W.P. Functional properties and nutritional quality of alkali- and heat-treated soy protein isolate. *Journal of Food Quality* **1999**, *22*, 119-133, doi:10.1111/j.1745-4557.1999.tb00545.x.
56. Savoie, L.; Parent, G.; Galibois, I. Effects of alkali treatment on the in-vitro digestibility of proteins and the release of amino acids. *Journal of the Science of Food and Agriculture* **1991**, *56*, 363-372, doi:10.1002/jsfa.2740560312.
57. Sarwar, G.; L'Abbé, M.R.; Trick, K.; Botting, H.G.; Ma, C.Y. Influence of feeding alkaline/heat processed proteins on growth and protein and mineral status of rats. *Advances in Experimental Medicine and Biology* **1999**, *459*, 161-177, doi:10.1007/978-1-4615-4853-9_11.
58. Laguna, L.; Picouet, P.; Guàrdia, M.D.; Renard, C.M.G.C.; Sarkar, A. In vitro gastrointestinal digestion of pea protein isolate as a function of pH, food matrices, autoclaving, high-pressure and re-heat treatments. *LWT - Food Science and Technology* **2017**, *84*, 511-519, doi:10.1016/j.lwt.2017.06.021.
59. Yin, S.-W.; Tang, C.-H.; Wen, Q.-B.; Yang, X.-Q.; Li, L. Functional properties and in vitro trypsin digestibility of red kidney bean (Phaseolus vulgaris L.) protein isolate: Effect of high-pressure treatment. *Food Chemistry* **2008**, *110*, 938-945, doi:10.1016/j.foodchem.2008.02.090.
60. Mariniello, L.; Giosafatto, C.V.L.; Di Pierro, P.; Sorrentino, A.; Porta, R. Synthesis and Resistance to in Vitro Proteolysis of Transglutaminase Cross-Linked Phaseolin, the Major Storage Protein from Phaseolus vulgaris. *Journal of Agricultural and Food Chemistry* **2007**, *55*, 4717-4721, doi:10.1021/jf0637269.
61. Tang, C.H.; Li, L.; Yang, X.Q. Influence of transglutaminase-induced cross-linking on in vitro digestibility of soy protein isolate. *Journal of Food Biochemistry* **2006**, *30*, 718-731, doi:10.1111/j.1745-4514.2006.00092.x.
62. Tang, C.-H.; Sun, X.; Yin, S.-W.; Ma, C.-Y. Transglutaminase-induced cross-linking of vicilin-rich kidney protein isolate: Influence on the functional properties and in vitro digestibility. *Food Research International* **2008**, *41*, 941-947, doi:10.1016/j.foodres.2008.07.015.
63. Li, Y.; Damodaran, S. In vitro digestibility and IgE reactivity of enzymatically cross-linked heterologous protein polymers. *Food Chemistry* **2017**, *221*, 1151-1157, doi:10.1016/j.foodchem.2016.11.044.

64. Sheng, W.W.; Zhao, X.H. Functional properties of a cross-linked soy protein-gelatin composite towards limited tryptic digestion of two extents. *Journal of the Science of Food and Agriculture* **2013**, *93*, 3785-3791, doi:10.1002/jsfa.6276.
65. Fu, M.; Zhao, X.H. Modified properties of a glycated and cross-linked soy protein isolate by transglutaminase and an oligochitosan of 5 kDa. *Journal of the Science of Food and Agriculture* **2017**, *97*, 58-64, doi:10.1002/jsfa.7682.
66. Zhu, C.Y.; Liu, H.F.; Fu, M.; Zhao, X.H. Structure and property changes of soybean protein isolates resulted from the glycation and cross-linking by transglutaminase and a degraded chitosan. *CYTA - Journal of Food* **2016**, *14*, 138-144, doi:10.1080/19476337.2015.1067646.
67. Gan, C.Y.; Cheng, L.H.; Azahari, B.; Easa, A.M. In-vitro digestibility and amino acid composition of soy protein isolate cross-linked with microbial transglutaminase followed by heating with ribose. *International Journal of Food Sciences and Nutrition* **2009**, *60*, 99-108, doi:10.1080/09637480802635090.
68. Yin, S.W.; Tang, C.H.; Wen, Q.B.; Yang, X.Q. Effects of acylation on the functional properties and in vitro trypsin digestibility of red kidney bean (Phaseolus vulgaris L.) protein isolate. *Journal of Food Science* **2009**, *74*, E488-E494, doi:10.1111/j.1750-3841.2009.01349.x.
69. El-Adawy, T.A. Functional properties and nutritional quality of acetylated and succinylated mung bean protein isolate. *Food Chemistry* **2000**, *70*, 83-91, doi:10.1016/s0308-8146(00)00079-0.
70. Achouri, A.; Zhang, W. Effect of succinylation on the physicochemical properties of soy protein hydrolysate. *Food Research International* **2001**, *34*, 507-514, doi:10.1016/s0963-9969(01)00063-1.
71. de Regil, L.M.; Calderón de la Barca, A.M. Nutritional and technological evaluation of an enzymatically methionine-enriched soy protein for infant enteral formulas. *International Journal of Food Sciences and Nutrition* **2004**, *55*, 91-99, doi:10.1080/09637480410001666478.
72. Budryn, G.; Zaczyńska, D.; Rachwał-Rosiak, D.; Oracz, J. Changes in properties of food proteins after interaction with free and β-cyclodextrin encapsulated hydroxycinnamic acids. *European Food Research and Technology* **2015**, *240*, 1157-1166, doi:10.1007/s00217-015-2419-9.
73. Rohn, S.; Petzke, K.J.; Rawel, H.M.; Kroll, J. Reactions of chlorogenic acid and quercetin with a soy protein isolate - Influence on the in vivo food protein quality in rats. *Molecular Nutrition and Food Research* **2006**, *50*, 696-704, doi:10.1002/mnfr.200600043.
74. Strauch, R.C.; Lila, M.A. Pea protein isolate characteristics modulate functional properties of pea protein–cranberry polyphenol particles. *Food Science and Nutrition* **2021**, *9*, 3740-3751, doi:10.1002/fsn3.2335.
75. Yang, J.; Bao, Z.; Wu, N.; Yang, X.; Lin, W.; Chen, Z.; Wang, J.; Guo, J. Preparation and characterisation of soya milk enriched with isoflavone aglycone fermented by lactic acid bacteria combined with hydrothermal cooking pretreatment. *International Journal of Food Science and Technology* **2015**, *50*, 1331-1337, doi:10.1111/ijfs.12784.
76. Chen, N.; Zhao, Q.; Sun, W.; Zhao, M. Effects of malondialdehyde modification on the in vitro digestibility of soy protein isolate. *Journal of Agricultural and Food Chemistry* **2013**, *61*, 12139-12145, doi:10.1021/jf404099y.
77. Rhee, K.S.; Rhee, K.C. Nutritional Evaluation of the Protein in Oilseed Products Heated with Sugars. *Journal of Food Science* **1981**, *46*, 164-168, doi:10.1111/j.1365-2621.1981.tb14555.x.
78. Wang, H.; Chen, Y.; Hua, Y.; Kong, X.; Zhang, C. Effects of phytase-assisted processing method on physicochemical and functional properties of soy protein isolate. *Journal of Agricultural and Food Chemistry* **2014**, *62*, 10989-10997, doi:10.1021/jf503952s.
79. Zhao, J.; Su, G.; Chen, C.; Sun, X.; Sun, W.; Zhao, M. Physicochemical and Structural Characteristics of Soybean Protein Isolates Induced by Lipoxygenase-Catalyzed Linoleic Acid Oxidation during in Vitro Gastric Digestion. *Journal of Agricultural and Food Chemistry* **2020**, *68*, 12384-12392, doi:10.1021/acs.jafc.0c02098.
80. Chen, N.; Zhao, M.; Sun, W. Effect of protein oxidation on the in vitro digestibility of soy protein isolate. *Food Chemistry* **2013**, *141*, 3224-3229, doi:10.1016/j.foodchem.2013.05.113.
81. Duque-Estrada, P.; Berton-Carabin, C.C.; Nieuwkoop, M.; Dekkers, B.L.; Janssen, A.E.M.; van der Goot, A.J. Protein Oxidation and In Vitro Gastric Digestion of Processed Soy-Based Matrices. *Journal of Agricultural and Food Chemistry* **2019**, *67*, 9591-9600, doi:10.1021/acs.jafc.9b02423.
82. Opazo-Navarrete, M.; Altenburg, M.D.; Boom, R.M.; Janssen, A.E.M. The Effect of Gel Microstructure on Simulated Gastric Digestion of Protein Gels. *Food Biophysics* **2018**, *13*, 124-138, doi:10.1007/s11483-018-9518-7.
83. Hall, A.E.; Moraru, C.I. Effect of High Pressure Processing and heat treatment on in vitro digestibility and trypsin inhibitor activity in lentil and faba bean protein concentrates. *LWT* **2021**, *152*, doi:10.1016/j.lwt.2021.112342.
84. Rui, X.; Fu, Y.; Zhang, Q.; Li, W.; Zare, F.; Chen, X.; Jiang, M.; Dong, M. A comparison study of bioaccessibility of soy protein gel induced by magnesiumchloride, glucono-δ-lactone and microbial transglutaminase. *LWT - Food Science and Technology* **2016**, *71*, 234-242, doi:10.1016/j.lwt.2016.03.032.
85. Ou, S.; Kwok, K.C.; Kang, Y. Changes in in vitro digestibility and available lysine of soy protein isolate after formation of film. *Journal of Food Engineering* **2004**, *64*, 301-305, doi:10.1016/j.jfoodeng.2003.10.013.
86. Omosebi, M.O.; Osundahunsi, O.F.; Fagbemi, T.N. Effect of extrusion on protein quality, antinutritional factors, and digestibility of complementary diet from quality protein maize and soybean protein concentrate. *Journal of Food Biochemistry* **2018**, *42*, doi:10.1111/jfbc.12508.
87. Wang, N.; Bhirud, P.R.; Tyler, R.T. Extrusion texturization of air-classified pea protein. *Journal of Food Science* **1999**, *64*, 509-513, doi:10.1111/j.1365-2621.1999.tb15073.x.
88. Li, S.; Jiang, Z.; Wang, F.; Wu, J.; Liu, Y.; Li, X. Characterization of rice glutelin fibrils and their effect on in vitro rice starch digestibility. *Food Hydrocolloids* **2020**, *106*, 105918, doi:10.1016/j.foodhyd.2020.105918.
89. Hu, B.; Chen, Q.; Cai, Q.; Fan, Y.; Wilde, P.J.; Rong, Z.; Zeng, X. Gelation of soybean protein and polysaccharides delays digestion. *Food Chemistry* **2017**, *221*, 1598-1605, doi:10.1016/j.foodchem.2016.10.132.
90. Huang, Z.; Gruen, I.; Vardhanabhuti, B. Intragastric Gelation of Heated Soy Protein Isolate-Alginate Mixtures and Its Effect on Sucrose Release. *Journal of Food Science* **2018**, *83*, 1839-1846, doi:10.1111/1750-3841.14192.
91. Khalesi, M.; Fitzgerald, R.J. In vitro digestibility and antioxidant activity of plant protein isolate and milk protein concentrate blends. *Catalysts* **2021**, *11*, doi:10.3390/catal11070787.
92. Baugreet, S.; Gomez, C.; Auty, M.A.E.; Kerry, J.P.; Hamill, R.M.; Brodkorb, A. In vitro digestion of protein-enriched restructured beef steaks with pea protein isolate, rice protein and lentil flour following sous vide processing. *Innovative Food Science and Emerging Technologies* **2019**, *54*, 152-161, doi:10.1016/j.ifset.2019.04.005.

93. Oñate Narciso, J.; Brennan, C. Whey and Pea Protein Fortification of Rice Starches: Effects on Protein and Starch Digestibility and Starch Pasting Properties. *Starch/Staerke* **2018**, *70*, doi:10.1002/star.201700315.
94. Sánchez-Vioque, R.; Vioque, J.; Clemente, A.; Pedroche, J.; Bautista, J.; Millán, F. Interaction of chickpea (Cicer arietinum L.) legumin with oxidized linoleic acid. *Journal of Agricultural and Food Chemistry* **1999**, *47*, 813-818, doi:10.1021/jf980889r.
95. Hsu, H.; Vavak, D.; Satterlee, L.; Miller, G. A multienzyme technique for estimating protein digestibility. *Journal of Food Science* **1977**, *42*, 1269-1273, doi:10.1111/j.1365-2621.1977.tb14476.x.
